# Procollagen I carboxy-terminal propeptide: a multi-faceted biomarker

**DOI:** 10.11613/BM.2026.020502

**Published:** 2026-04-15

**Authors:** Sharon Philip Jain, Niharika Ikkurthy, Swetha Sundara, Shervinchris Philip Jain

**Affiliations:** 1 Department of Medicine, David Tvildiani Medical University, Tbilisi, Georgia; 2 Smidt Heart Institute, Cedars-Sinai Medical Center, Los Angeles, USA; 3 Department of Community Medicine, Rangaraya Medical College, Kakinada, India; 4 Department of Medicine, Jubilee Mission Medical College and Research Institute, Kerala, India

**Keywords:** Procollagen I carboxy-terminal propeptide, collagen, biomarker, fibrosis

## Abstract

Procollagen I carboxy-terminal propeptide (PICP) is a cleavage product of procollagen I, the precursor of type I collagen. Type I collagen is the most abundant type of collagen in the human body and plays a central role in maintaining structural integrity across tissues such as skin, bone, vasculature, and connective organs. Procollagen I carboxy-terminal propeptide, released during collagen synthesis, has been associated with fibrotic activity and tissue remodeling in different conditions across organ systems, including the heart, lungs, liver, kidneys, skin, and musculoskeletal system. Although many studies have investigated PICP in individual diseases, ranging from cardiomyopathies to liver cirrhosis and chronic lung disorders, its overall diagnostic and prognostic value remain incompletely defined. Serum PICP is a biomarker of fibrotic activity, with potential to be a valuable screening tool or component of diagnostic risk scores before moving to invasive methods such as biopsy. The ability to monitor fibrotic progression or therapeutic response through a simple blood test presents an opportunity for earlier diagnosis, improved disease stratification, and more efficient treatment monitoring. This literature review aims to provide a comprehensive synthesis of current knowledge on PICP, focusing on its biological role in collagen metabolism and clinical applications. By critically evaluating recent advances in assay methodologies, reference ranges, and interpretation in diverse patient populations, this review highlights both the strengths and limitations of PICP as a diagnostic and prognostic tool to identify future directions for research and clinical practice.

## Graphical abstract

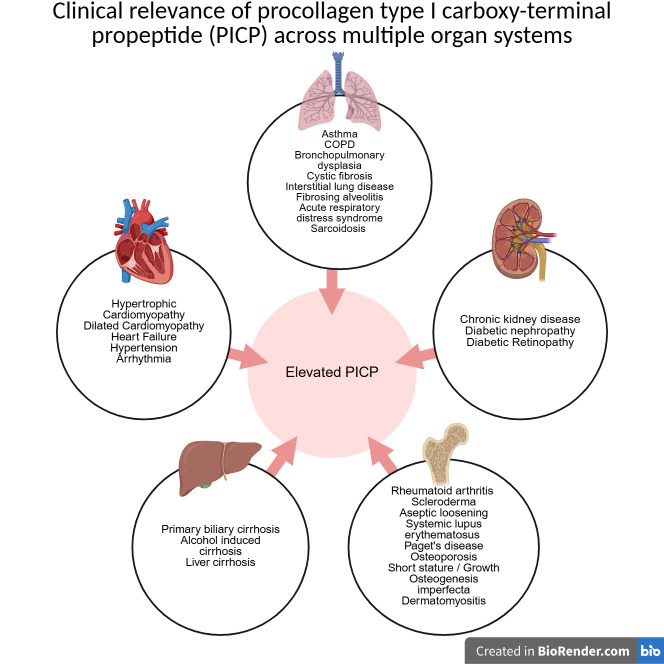

## Highlights

• Procollagen I carboxy-terminal propeptide (PICP) is a marker of collagen production across many body systems

• It helps assess prognosis and monitor treatment across several diseases

• Emerging evidence suggests PICP could become a valuable tool across medical specialties

• Further research is required to define its role in routine diagnostics

• More studies are needed to establish PICP reference values

## Introduction

Collagen is an extracellular structural protein whose function depends upon a triple helical structure of three polypeptide chains twisted around each other, wherein there are additional globular domains in addition to the triple helices (*1*, *2*). Among the 28 known collagen types, type I collagen (COL1) is the most abundant in tissues such as skin, bone, tendons, and blood vessels, accounting for approximately 90% of the body’s collagen content (*3*, *4*).

Due to its near ubiquity, the monitoring of collagen synthesis can play an important diagnostic role in multiple disease systems (*2*, *5*, *6*). Often, propeptides are used as a measure of collagen synthesis and degradation, with serum concentrations of carboxy-terminal (C-terminal) propeptide of procollagen I (PICP) used as a measure of extracellular COL1 synthesis, and C-terminal telopeptide of type I collagen (ICTP) used as a marker of extracellular COL1 degradation (*5*).

Since 1974, PICP has been quantified using immunoassays, and numerous biomarker utilities have been determined over the decades (*7*). Given its widespread distribution and structural role, dysregulation of COL1 metabolism is associated with a range of pathological conditions (*2*). Diagnostic uses have been discovered in wound healing, liver disease, cancer monitoring, heart disease, inflammatory bowel disease, interstitial lung disease, and many other disease processes (*8*-*13*).

This literature review synthesizes nearly five decades of research on PICP, providing an overview of its structure, biological mechanisms, and clinical relevance. By evaluating the strengths, limitations, and current applications as a diagnostic and prognostic biomarker, this review aims to objectively assess PICP’s potential role in clinical practice and acknowledge that future research may provide additional insight and expand our existing knowledge of it.

## Materials and methods

A comprehensive literature search was conducted across databases PubMed, Google Scholar, Embase, and Scopus from inception to May 2025. Search terms included “Procollagen type I C-terminal propeptide”, “PICP”, combined with keywords related to its clinical role, such as “diagnosis”, “prognosis”, “fibrosis”, “biomarker”, and systemic keywords, such as “cardiology”, “oncology”, “pediatric”, “dermatology”, “bone”, “hepatic”, “respiratory”, “neurology” and “renal”, as well as suitable modifiers. Clinical studies reporting on the role of PICP in diagnosis, prognosis, or disease monitoring in humans were eligible for inclusion. Case reports, editorials, conference abstracts and animal studies were excluded.

## Structure and physiological role of procollagen I C-terminal propeptide

**Figure 1:**
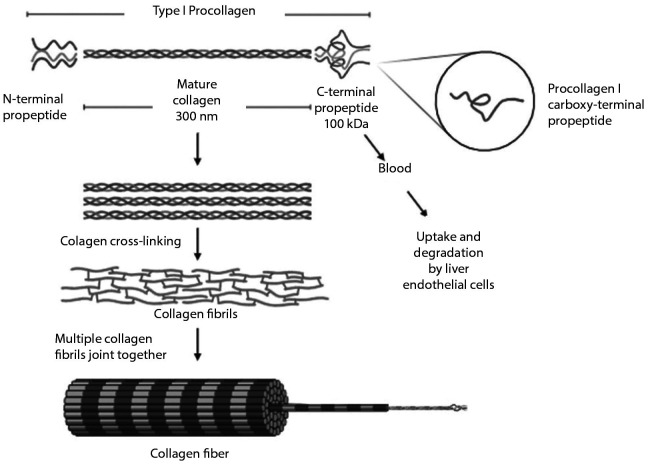
Schematic representation of type I collagen synthesis and release of procollagen type I carboxy-terminal propeptide (PICP). Type I procollagen is synthesized with N- and C-terminal propeptides. While the N-terminal is removed during maturation, the C-terminal propeptide is cleaved and released into the blood and later degraded by liver endothelial cells. Mature collagen is formed from the cross-linking of fibrils. Figure created using BioRender.com.

Procollagen I C-terminal propeptide is a terminal component of the precursor molecule of COL1, with the other terminal component being procollagen I nitro-terminal (N-terminal) propeptide (PINP). Procollagen I is synthesized within the endoplasmic reticulum, and the two terminal propeptides serve to prevent premature fiber formation. After the molecule reaches the extracellular space, the C-terminal and N-terminal parts of the molecule split from the collagen molecule during the process of procollagen conversion to collagen (Figure 1) (*14*, *15*). The splitting of PICP from procollagen is achieved by procollagen C-peptidases, after which the mature collagen molecules spontaneously assemble into stable fibers (*6*). Because PICP is produced in a 1:1 stoichiometric ratio to COL1, with each cleaved C-terminal propeptide molecule equivalent to one collagen molecule produced, its serum concentrations are a reliable measure of collagen activity and production (*16*).

Due to its molecular weight (100 kDa), PICP is not affected by glomerular filtration, and its clearance occurs primarily through uptake by the liver’s sinusoidal endothelial cells, making its clearance independent of renal factors (*17*, *18*). It is important to distinguish PICP from other C-telopeptides, such as ICTP. While PICP reflects collagen synthesis, ICTP is a marker of collagen degradation, released during its breakdown and tissue remodelling, and exists between the terminal propeptides and the mature collagen molecule (*3*, *19*).

## Methods of determination

In most of the studies on PICP, serum is the primary matrix used, with the occasional use of plasma or tissue samples. Units are typically expressed in ng/mL or µg/L; a lack of consistency across the studies limit comparisons. Methods used for analysis include enzyme-linked immunosorbent assays (ELISA) and older radioimmunoassays (RIA). In earlier works using RIA, preliminary reference intervals for healthy adults (18-61 years old) were deduced as 38-202 µg/L for men and 50-170 µg/L for women (*4*). An ELISA assay for PICP with a detection limit of 1 ng/L, measurable range of 1-640 ng/mL, and < 10% intra- and inter-assay imprecision values was developed. The result was an efficient method to produce PICP-specific antibodies, enabling potential use in clinical diagnostic applications (*20*). Despite these advances, differences in calibrators and variabilities in commercial kit standards act as barriers to interpretation and clinical implementation (*21*). Within ELISA methods, cut-offs vary from assay to assay, and units are inconsistently reported. Other limitations include large sample volumes and long incubation times required by traditional PICP assays (*20*). This is a barrier to consistent interpretation and impedes accurate pooling of results to understand the biomarker and its true normal range. Although few studies have validated PICP for long-term monitoring, the absence of standardized reference ranges limits its applicability. Longitudinal use of PICP is further complicated by inter-individual differences and potential confounding factors such as hepatic dysfunction and medication use (*22*-*24*). Immunoassays dominate PICP measurement today, especially ELISA, chemiluminescence immunoassay, and electrochemiluminescence immunoassay. Radioimmunoassays and immunoradiometric assay are historically important but have now been largely phased out due to radioactivity concerns, whereas high-performance liquid chromatography is mainly research-oriented and not used clinically (*4*, *20*). Though PICP presents itself as a promising biomarker of collagen type I synthesis and fibrosis, its clinical utility is limited by assay variability, lack of harmonized reference ranges, and confounding systemic factors. Until these issues are resolved, PICP remains more of a research tool than a widely adopted diagnostic marker.

## Clinical utility and diagnostic value of procollagen I C-terminal propeptide in different systemic diseases

### Cardiovascular disease

Associations between PICP and cardiovascular disease have been studied for over two decades, with one of the initial connections being made between collagen scar formation after acute myocardial infarction (*25*). Following studies identified the role of PICP in hypertensives, where PICP was found to be a more accurate predictor of myocardial fibrosis compared to left ventricular mass index and E/A ratio (E-wave/A-wave ratio, an echocardiographic marker of left ventricular diastolic function), and a valuable tool for measuring the cardioprotective effects of antihypertensive medications (*5*, *11*, *26*). This same association makes PICP a useful biomarker of myocardial fibrosis in hypertrophic cardiomyopathy, with a strong correlation existing between plasma PICP concentrations and myocardial PICP content (*27*).

Procollagen I C-terminal propeptide was also found to be a sensitive and specific testing metric for detecting left ventricular ejection fraction (LVEF) heart failure (HF), with 86.2% sensitivity and 98.8% specificity in distinguishing between normal individuals and those with HF (*28*). Therefore, PICP can act as an additional tool for confirmatory diagnosis alongside more established biomarkers of heart failure, such as N-terminal pro-B-type natriuretic peptide (NT-proBNP), and offer insight into myocardial damage rather than the pressure or volume overload (*28*). Elevated serum PICP was also found to have a correlation with post-surgical atrial fibrillation in patients after cardiac surgery, which may be explained by the presence of increased left atrial myocardial fibrosis found in those with elevated PICP concentrations (*29*, *30*).

In recent years, PICP has also been found to play a role in dilated cardiomyopathy (DCM). Studies have shown that an analysis of combinations of matrix metallopeptidase 1, PICP, and other serum biomarkers provides enough insight into DCM for risk stratification. The presence of higher concentrations of serum PICP was also associated with higher rates of adverse outcomes in those with DCM (*31*).

Baseline PICP concentrations were found to be higher in patients with Friedreich’s ataxia than in the general population and predictive of left ventricular dilation over time, suggesting myocardial fibrosis leading to cardiomyopathy and arrhythmias (*32*). Overall, although PICP is unlikely to serve as a standalone biomarker for cardiovascular disease, it holds potential, pending further validation, to become an integral component of multi-marker diagnostic scores for risk assessment and personalized cardioprotective therapy.

### Bone and connective tissue disease

Women with rheumatoid arthritis had significantly reduced PICP due to the negative impact of chronic inflammation on bone formation and remodeling (*33*). Subsequent studies examined the effects of hormone replacement therapy on bone biochemical markers such as PICP in postmenopausal women with rheumatoid arthritis. Further, they corroborated the results with the Larsen score to check if it is predictive of bone mineral density (BMD) (*34*, *35*). The decrease in PICP at 1 and 2 years follow-up, a consequence of reduced need for bone turnover, was associated with improved BMD and a good Larsen score; these findings reinforced previous similar studies (*34*-*36*).

Synovial fluid and suction blister fluid PICP concentrations are increased in systemic lupus erythematosus and scleroderma patients (*37*-*39*). Elevated concentrations of PICP are observed in systemic sclerosis, with higher concentrations in the diffuse type involving viscera. Procollagen I C-terminal propeptide can be used as a marker of severity and determinant of prognosis and risk of pulmonary fibrosis (*40*, *41*). Dermatomyositis patients have significantly elevated serum PICP, which was correlated with increased creatine kinase levels, indicative of active muscle damage (*42*). These findings suggest that PICP can be potentially used as a follow-up marker to monitor treatment efficacy and stratify severity in various rheumatologic diseases.

Beyond autoimmune diseases, PICP can offer insight into orthopedic conditions. The conventional methods of X-ray are widely used and straightforward, whereas techniques like periprosthetic BMD measurements can be more technically challenging and often detect aseptic loosening (AL) in total hip arthroplasty only at a late stage. Whether collagen biomarkers can identify individuals at risk of AL at early stages, since biochemical changes in bone turnover can be detected before radiographic signs of loosening, was investigated. He *et al.* concluded that an elevation in PICP concentrations as a means of compensation can be an early indicator of altered bone turnover in patients with prosthetic implants, suggesting potential loosening (*43*). Serum PICP concentrations were also identified as a sensitive diagnostic indicator of skull involvement in Paget’s disease (*44*).

Beyond these conditions, due to the role of PICP in bone formation, it has been extensively studied in osteoporosis, with PICP being found to be a predictor of osteoporotic fracture (*45*). Procollagen I C-terminal propeptide release is directly related to the rate of bone collagen synthesis and can be used as a proxy measure of osteoblast activity (*46*). Earlier studies have shown that PICP concentrations decrease in response to antiresorptive therapy, such as estrogen, and increase in response to anabolic therapy, such as teriparatide. Correlation with BMD was also identified for PICP, hence making it useful to monitor treatment response compared to imaging-based approaches, which require a longer time period to pick up alterations in BMD (*47*-*49*). Though PICP concentrations are similar in perimenopausal and menopausal women, it is useful in distinguishing women with higher turnover (lower PICP) and women with low turnover (higher PICP) amongst menopausal women (*47*). The former group is at increased risk of osteoporosis and thus can be classified as potentially higher risk and could benefit from earlier initiation of hormone replacement therapy or calcium supplements. However, despite these positive results, when compared to other bone formation markers such as PINP, PICP was found to be inferior (*21*, *50*). Though PICP provides insight into bone metabolism and connective tissue diseases, it cannot be considered a definitive and sole diagnostic measure. It may be a useful tool when used alongside PINP and imaging. Future work should focus on integration to improve clinical applicability.

### Oncology

Given its role in bone formation, PICP has potential utility in oncology as a biomarker to detect and monitor bone metastases (BM). Procollagen I C-terminal propeptide reflects the rate of type I collagen synthesis and may parallel the increased bone turnover lesions seen in metastases. Prostate-specific antigen (PSA), while commonly used, lacks specificity for prostate cancer (PC). Procollagen I carboxy-terminal propeptide can act as an adjunct tool to diagnose early skeletal metastases of prostate adenocarcinoma and improve overall specificity (*51*-*53*). Procollagen I C-terminal propeptide and PSA concentrations were significantly higher in PC patients with BM compared to those with benign prostate hyperplasia (*46*). Lung and breast cancer (BC) are two other cancers commonly associated with BM. However, studies in BC patients had conflicting results. Two studies showed PICP is not nearly as sensitive as other collagen biomarkers in diagnosing metastasis in BC, limiting its clinical efficacy as a standalone marker (*54*, *55*). Whereas Song *et al.* demonstrated statistically significant results, PICP concentrations were higher in the BC bone metastases group, and a decline was observed following 3 months of treatment. They concluded that PICP can be used to diagnose and assess treatment efficacy in BC metastases (*56*).

Though not specific (2.1%), PICP was highly sensitive (95.2%) in detecting BM in lung cancer patients. While it cannot replace existing superior standard modalities, it may be a valuable adjunctive tool, especially in resource-limited settings (*57*-*59*). While PICP alone was insignificant in women with epithelial ovarian cancer, a low PICP:PINP ratio was associated with faster metastatic progression and poor clinical outcome (*60*). Overall, although elevated PICP has been linked to BM in cancer, it lacks adequate specificity to be used alone. Nevertheless, it may improve clinical assessment and support early detection of skeletal involvement in prostate cancer, monitoring treatment response in breast cancer, and identify and assess metastatic progression in lung and ovarian cancer when combined with other routine imaging and biomarkers.

### Hepatic disease

End-stage liver disease and liver cirrhosis are marked by increased collagen deposition triggered by inciting factors such as chronic alcohol use or hepatitis infections, leading to fibrosis, the precursor to cirrhosis (*22*, *23*). Liver biopsy is considered to be the gold standard modality to evaluate the severity of fibrosis, but as it is an invasive tool, it may not be ideal for all patients or to monitor disease course on a routine basis, therefore, research has been conducted on the utility of alternative non-invasive collagen markers (*23*).

Two such frequently investigated markers are PICP and procollagen type III C-terminal propeptide (PIIICP). Though PIIICP is widely used as a collagen turnover biomarker, PICP offers the advantage of existing in a uniform form in the serum, without any variation, and is metabolized entirely through the liver *via* specific receptors and therefore remains unaffected in patients with co-existing renal diseases (*61*, *62*). Studies on PICP were predominantly in chronic alcoholic patients and are limited in number. Studies demonstrated the presence of maximally increased PICP concentrations in chronic alcoholic patients with hepatitis and cirrhosis and adequately elevated concentrations in patients with fatty liver and non-alcoholic diseases such as primary biliary cirrhosis (*23*, *61*, *62*). A positive correlation was noted between high levels of hepatic inflammation and the severity of fibrosis with PICP (*23*, *61*).

Lower concentrations of this biomarker cannot be used to exclude hepatic disease definitively; instead, they are best utilized as adjunctive tools in patients where liver biopsy is not feasible, and can also help monitor therapeutic response when baseline values are elevated (*23*). Procollagen type III N-terminal propeptide (PIIINP) is generally considered a more sensitive marker for early fibrotic changes and better reflects initial disease burden than PICP, which explains its preference as a biomarker (*62*). Considering the drawbacks of both PIIINP and PICP and that studies have shown that evaluating the ratio of PIIINP to PICP correlates well with both clinical and morphological severity of liver disease, rather than relying solely on PICP or PIIINP, it is more rational to focus research efforts on the PIIINP/PICP ratio as a means of assessing the extent and severity of hepatic fibrosis (*23*, *62*). In hepatic disease, PICP shows promise as a non-invasive marker of fibrosis, especially in alcoholic liver disease and cirrhosis, but current studies are limited and require further validation.

### Renal disease

The role of PICP in renal disease is often related to its role in other disease systems. Procollagen I carboxy-terminal propeptide in pre-dialysis chronic renal failure is a possible noninvasive indicator of bone turnover in both adults and children (*24*, *63*). It is elevated in those with diabetes, but the rise in serum PICP is associated with the concurrent presence of hypertension. Procollagen I carboxy-terminal propeptide is also an indicator of potential diabetic complications, such as diabetic retinopathy or nephropathy, suggesting that PICP may reflect early microvascular damage and fibrotic progression (*64*).

Procollagen I carboxy-terminal propeptide is an active indicator of a pro-fibrotic state, with elevated concentrations of the biomarker being associated with subclinical fibrosis and subclinical renal disease (*65*, *66*). In addition, PICP is a good predictor of all-cause mortality in advanced kidney disease, with one univariate analysis showing PICP above a cut-off point (PICP > 297.31 µg/L) being associated with a five-fold increased risk of mortality from chronic kidney disease (HR 5.07, 95% confidence interval (CI) 1.94-13.29, *P* = 0.001) (*65*, *67*). Koh *et al.* also found significant associations between PICP and chronic renal failure (*68*). Renal and cardiac disease often go hand in hand, and elevated PICP may be an indicator of concurrent conditions and an interplay of two disease processes affecting one another (*65*, *66*, *68*). Clinically, PICP could be monitored longitudinally in high-risk patients such as those with diabetes, severe hypertension, or advanced renal disease to detect early fibrotic changes and assess therapeutic effectiveness. As a non-invasive marker of subclinical renal damage, it can help provide prognostic insight into long-term outcomes and a basis for future clinical studies.

### Respiratory disease

Airway remodeling, characterized by predominantly COL1 deposition, drives the pathophysiology of chronic obstructive pulmonary disease (COPD) and asthma, leading to airway narrowing (*69*, *70*). Zeng *et al.* investigated whether serum PICP concentrations were associated with the degree of inflammation in stable COPD patients and concluded that the concentrations were significantly higher in COPD cases (*69*). They hypothesized that a higher level could potentially translate to a poorer prognosis and increased severity, though further studies are needed to draw conclusive results (*69*). Another study involving asthma patients measured PICP concentrations in the sputum following inhaled steroids, and a decrease in the collagen biomarker was noted (*70*). The above cases suggest using PICP as a biomarker to assess the degree of ongoing inflammation and airway remodeling changes in obstructive lung diseases and identify individuals more at risk of exacerbations.

Given its role in collagen synthesis, it is logical to study its concentrations in granulomatous and interstitial diseases such as sarcoidosis, which causes progressive pulmonary fibrosis. A cross-sectional study comparing serum PICP and bronchoalveolar lavage fluid (BALF) found that the latter was a better and more sensitive indicator of pulmonary sarcoidosis. However, the concentrations were not corroborated by the standard modalities, such as serum angiotensin converting enzyme and chest radiography, and the author attributed the discrepancy to the cross-sectional nature of the study. In contrast, another study established no association between the two (*71*, *72*). More studies are warranted on this topic.

Fibrosing alveolitis is another progressive lung disease with a poor prognosis, and no reliable predictors of progression have been established yet. One study concluded that BALF PICP concentrations are increased in this disease and can be used to assess pulmonary involvement and demonstrated a negative correlation between BALF and epithelial lining fluid (ELF) PICP and diffusing capacity of the lungs for carbon monoxide, indicating that higher concentrations of PICP imply a decreased efficiency in the transfer of gases (*73*). Another study demonstrated higher BALF and ELF PICP concentrations in asbestos patients with parenchymal involvement, suggesting its use as a biomarker to identify the parenchymal participation in those individuals exposed to asbestos (*74*). The role of PICP was studied in acute respiratory distress syndrome/acute lung injury subjects. Procollagen I carboxy-terminal propeptide was elevated in these subjects, and the balance of collagen turnover was more in favor of synthesis, promoting fibrotic changes early in the disease course. Higher concentrations could potentially mean higher degrees of fibrotic changes and poorer outcomes with increased mortality, necessitating the need for more interventions (*75*). Given its correlation with fibrotic activity and impaired gas exchange, studies have shown that PICP can be used as a biomarker to assess disease severity and prognosis in conditions such as fibrosing alveolitis, sarcoidosis, ARDS, *etc.* Elevated BALF PICP concentrations correlate with the extent of fibrosis and reduced diffusing capacity for carbon monoxide (DLCO), suggestive of both structural and functional impairment. However, current evidence is limited, so it cannot replace gold standard procedures (spirometry, chest X-ray, lung biopsy, *etc*). Measurement of BALF PICP can be offered as a practical alternative for patients with fibrotic lung disease where lung biopsy is contraindicated, for those who decline biopsies, or to monitor disease progression when repeated biopsies aren’t practical.

### Pediatric disease

Dexa-scan and quantitative computed tomography are the gold standards for evaluating BMD due to their accuracy and low radiation exposure. However, in a pediatric setting, they are associated with challenges such as irritability, crying, and discomfort, leading to inconsistent results and not capturing real-time changes in bone metabolism. In contrast, bone turnover markers such as PICP are minimally invasive. Patients can be monitored over time, reflecting the current state of bone metabolism, making the biomarker valuable for pediatric patients (*76*, *77*). Type I and III collagen biomarkers, especially PICP, reflect the growth velocity in the pediatric age group, and the concentrations are comparatively lower in adults (*78*-*81*). In this context, PICP serves not only as a biomarker for bone health but also as an indicator of somatic growth.

Multiple studies have investigated the potential role of PICP as a biomarker to diagnose osteogenesis imperfecta (OI) prenatally. While the lowest concentrations are seen with type 4 OI, abnormally low concentrations were seen in the other types, and a positive correlation was established between the severity of OI and PICP (*78*, *82*). It is indicative of the role of PICP in disease stratification.

Procollagen I carboxy-terminal propeptide has shown promise in evaluating the response to treatments in conditions causing reduced BMD or stature. Since low vitamin K has been implicated in reduced BMD in children with cystic fibrosis (CF), the concentrations of bone turnover markers, such as PICP, before and after vitamin K administration in cystic fibrosis patients with defined low BMD was assessed (*83*). An increase in PICP, reflecting collagen biosynthesis, was observed following vitamin K treatment, concluding that long-term vitamin K supplementation may ultimately improve BMD scores in CF patients. Procollagen I carboxy-terminal propeptide can be potentially used as a marker to evaluate response to vitamin K and monitor BMD (*83*-*85*). A 3-year follow-up of prepubertal children with idiopathic short stature (ISS) treated with growth hormone (GH) therapy showed improvement in BMD and a gradual rise in PICP concentrations, reaching control numbers by the end of the study (*86*). Thus, it can be used to measure the effectiveness of long-term GH therapy in patients with ISS or GH-deficient individuals and help diagnose GH-deficient individuals (*87*-*89*).

In individuals with low BMD due to malabsorptive diseases such as celiac disease, PICP can be measured to check the effectiveness of a gluten-free diet in normalizing BMD (*90*). Procollagen I carboxy-terminal propeptide was negatively correlated with gestational age (GA) and birth weight in studies involving appropriate-for-gestational-age (AGA) neonates, implying that those born earlier with lower GA and weight have higher bone turnover, which may be harmful to bone health (*91*-*93*). However, similar concentrations were observed in small for gestational age (SGA) infants when concentrations lower than AGA infants were expected; reduced bone mineral content rather than defective collagen metabolism was proposed as the reason (*94*). Procollagen I carboxy-terminal propeptide was also found to be possibly affected by the baby’s gender, maternal health, and parity in some studies, whereas others found no association (*92*, *95*).

Furthermore, maternal health conditions may indirectly influence neonatal PICP. Insulin-like growth factor-1 (IGF-1) acts as a growth regulator, and maternal conditions such as preeclampsia reduce IGF-1, reducing PINP concentrations. Based on this fact, Kanjantie *et al.* proposed that cord blood PINP may serve as valuable markers for both fetal growth and maturity, and could potentially be used to predict postnatal growth rates in preterm infants (*93*). Procollagen I carboxy-terminal propeptide may also serve as an early indicator of fibrotic complications in neonatal conditions. It was identified as a reliable predictor for bronchopulmonary dysplasia in low-birth-weight infants (*96*). Posthemorrhagic hydrocephalus (PHHC) is a complication seen in preterm infants following intraventricular hemorrhage (IVH). Posthemorrhagic hydrocephalus babies were observed to have high PICP concentrations compared to non-hemorrhagic hydrocephalus babies, denoting early fibrotic changes in the former case. It was inferred that higher PICP following IVH increase the risk for PHHC (*97*). Procollagen I carboxy-terminal propeptide provides a window into pediatric health by reflecting skeletal growth and fibrotic complications, presenting itself as a promising tool to monitor bone and developmental disorders in early life.

### Other clinical associations of procollagen I carboxy-terminal propeptide

Collagen plays an important role in maintaining the structural integrity of skin by being involved in tissue remodeling and fibrosis. In dermatological conditions characterized by excessive collagen deposition, such as systemic sclerosis and scleroderma, serum PICP can be a measure of the severity of skin pathologies (*37*, *41*, *98*). Its minimally invasive feature offers a practical advantage over skin biopsies.

Beyond dermatology, PICP is also significant in neurological injury. Subdural PICP concentrations were significantly elevated following head trauma-induced subarachnoid hemorrhage, indicating increased collagen synthesis and fibrosis during the healing process. Thus, higher PICP concentrations may be associated with an increased risk of complications such as chronic subdural hematomas, effusions, or late posthemorrhagic hydrocephalus, suggesting that it can be potentially used to identify patients at risk of developing chronic fibrotic sequelae following neurological trauma (*99*, *100*). Overall, PICP shows promise as a cross-disciplinary biomarker for monitoring fibrosis in both dermatological and neurological conditions.

### Summary of evidence

Procollagen I carboxy-terminal propeptide has a clearly established biological basis as a marker for type I collagen synthesis; however, its clinical utility varies across systems. Evidence is strongest in cardiovascular remodelling, bone and connective tissue disorders, metastatic surveillance, and chronic renal disease. In these settings, PICP concentrations correlate with fibrotic burden, disease severity, and adverse outcomes but remain limited by specificity and performance compared to other markers (*e.g.*, PINP). In contrast, evidence remains limited in hepatic and pediatric diseases, as interpretation is confounded by clearance mechanisms and physiological collagen turnover. Despite these constraints, overall across systems, PICP is best positioned as an adjunct biomarker, useful for monitoring disease progression and treatment response, and fine-tuning risk stratification, rather than as a definitive diagnostic test.

## Limitations and challenges

The widespread presence of PICP is a double-edged sword, as the biomarker is elevated in a wide range of diseases, yet often lacks specificity for the same reason (*19*). Elevations may occur in cardiovascular, hepatic, and renal diseases, among others, making it difficult to attribute the elevated PICP to a single etiology, especially in the presence of multiple comorbidities. The mechanism by which PICP is cleared from the body may also obscure the presence of disease. In the presence of hepatic disease, the impaired clearance of PICP causes increased serum concentrations of the biomarker regardless of the rate of collagen turnover. In such states, elevations due to other concurrent disease processes, such as heart failure or chronic kidney disease, are masked by the already elevated serum concentrations of PICP (*63*, *67*). Similarly, increases in PICP may reflect general bone turnover rather than metastasis alone, or in children, it could be part of physiologic growth development (*46*, *79*). These examples demonstrate how, sometimes, elevations in PICP could be due to non-pathological processes.

## Future potential and perspectives

Procollagen I carboxy-terminal propeptide is now being used in combination with imaging strategies such as cardiac magnetic resonance imaging with late gadolinium enhancement to improve prognostic power in conditions such as aortic stenosis, dilated cardiomyopathy, and heart failure (*101*, *102*). This layering of diagnostic tools may help in more accurate risk stratification, prognostic estimates, and precision medicine. Exploration of ratios such as PIIINP/PICP ratio should be investigated in other key systemic diseases, such as cardiovascular and renal diseases, in the hopes that it may also improve specificity and prognostic value, as observed in hepatic fibrosis. Beyond diagnosis and prognosis, PICP also holds potential as a tool for therapeutic monitoring, especially in conditions where stabilizing or reversing fibrosis is the target. As discussed in this review, across cardiac, hepatic, respiratory diseases, *etc*., evidence suggests that changes in PICP concentrations parallel the responses to anti-fibrotic therapies, reflective of reduced fibrotic activity. This makes PICP a minimally invasive option to track therapeutic effectiveness over time and enables earlier detection compared to imaging methods. To counteract the challenges to standardization, mass spectrometry may be a new method of measuring PICP. While currently used in the context of research, the method may offer the added benefit of lending PICP better specificity. Mass spectrometry is currently a common practice for other biomarkers, such as PINP and procollagen type III biomarkers (*103*). Larger-scale studies on PICP must be done, looking at a broader range of diseases. Fibrosis occurs in numerous diseases across organ systems, and the diagnostic role of PICP should be evaluated, with disease-specific cutoffs to aid clinical decision-making and reasoning. Furthermore, the relationships between PICP and other diagnostic techniques and biomarkers must be explored.

## Conclusion

Procollagen I carboxy-terminal propeptide is a promising biomarker of type I collagen synthesis with potential utility across multiple disease processes. Its characteristic of being present in a 1:1 stoichiometric ratio with COL1 production makes it an accurate and reliable measure of collagen synthesis. Coupled with its clearance independent of renal function, it becomes a potentially reliable tool in clinical practice. Due to confounding factors in clinical practice, elevated PICP concentrations should be interpreted in the context of underlying comorbidities and age, rather than as a standalone indicator.

This review highlights the diverse diagnostic and prognostic potential of PICP in several disease systems and patient populations. Evidence of PICP is most compelling and interesting in renal disease, where it predicts all-cause mortality in advanced kidney disease. Though cardiovascular and bone disorders have potential in conditions such as heart failure and monitoring treatment in osteoporosis, PICP is typically combined with other routine biomarkers and imaging, limiting its independent use here. Oncology, pediatrics, respiratory diseases, and other specialties remain of academic interest, reflecting collagen turnover without sufficient clinical evidence yet. Overall, PICP can contribute to risk stratification, disease monitoring, and severity assessment. Its integration into multimodal diagnostic strategies may yield meaningful clinical benefits. Future work must focus on assay standardization, disease-specific reference ranges, and large-scale longitudinal studies to validate existing findings. With these advances, PICP could evolve into a valuable adjunct in routine diagnostics and precision medicine.

## Data Availability

No data was generated during this study, so data sharing statement is not applicable to this article.
